# High prevalence of human T-lymphotropic virus 2 (HTLV-2) infection in villages of the Xikrin tribe (Kayapo), Brazilian Amazon region

**DOI:** 10.1186/s12879-019-4041-0

**Published:** 2019-05-22

**Authors:** Isabel Luís Jocene Braço, Keyla Santos Guedes de Sá, Mishelle Waqasi, Maria Alice Freitas Queiroz, Andréa Nazaré Rangel da Silva, Izaura M. V. Cayres-Vallinoto, Sandra Souza Lima, Marluísa de Oliveira Guimarães Ishak, Ricardo Ishak, João Farias Guerreiro, Antonio Carlos Rosário Vallinoto

**Affiliations:** 1 Laboratory of Virology, Institute of Biological Sciences, Federal University of Pará, Belém, Para Brazil; 2 Department of Life Sciences, Keele University, Keele, UK; 3 Laboratory of Human and Medical Genetics, Institute of Biological Sciences, Federal University of Pará, Belém, Para Brazil; 4 Programa de Pós-Graduação em Biologia de Agentes Infecciosos e Parasitários, Universidade Federal do Pará, Belém, Para Brazil

**Keywords:** HTLV-2, Xikrin, Kayapo, Brazilian Amazon

## Abstract

**Background:**

Studies have shown that the human T-lymphotropic virus 2 (HTLV-2) is endemic in several indigenous populations of the Brazilian Amazon and molecular analyses have shown the exclusive presence of HTLV-2 subtype 2c among the indigenous groups of this geographical region.

**Methods:**

The present study characterizes the prevalence of HTLV-2 infection in three new villages of the Xikrin tribe, in the Kayapo group, according to their distribution by sex and age. The study included 263 samples from individuals from the Kateté, Djujeko and Oodjã villages. Plasma samples were tested for the presence of anti-HTLV-1/2 antibodies using enzyme-linked immunosorbent assays (ELISA). Seropositive samples were confirmed using real-time PCR, nested PCR and sequencing.

**Results:**

The serological and molecular results confirmed the sole presence of HTLV-2 in 77 (29%) samples, with a prevalence of 38% among women and 18% among men. In these communities, it was found that the prevalence of HTLV-2 infection increased with age. Nucleotide sequences (642 bp, 5’LTR) from eight samples were subjected to phylogenetic analysis by the neighbor-joining method to determine the viral subtype, which confirmed the presence of HTLV-2c.

**Conclusions:**

The results of the present study establish the presence of HTLV-2 infection in three new villages of the Xikrin tribe and confirm the high endemicity of the infection in the Kayapo indigenous group of the Brazilian Amazon.

## Backgrounds

The human T-lymphotropic viruses 1 and 2 (HTLV-1 and HTLV-2), members of the family *Retroviridae,* are retroviruses with tropism for T lymphocytes [1, 2]. HTLV-1 and HTLV-2 are believed to have been disseminated along human migratory pathways from Africa to Europe, Asia and the Americas, and they are currently distributed worldwide [3–10].

HTLV-2 is endemic in several African populations [8, 9] and in indigenous groups in North, Central and South America [6, 11–27]. Molecular studies have shown the existence of four molecular subtypes of HTLV-2: HTLV-2a, HTLV-2b, HTLV-2c and HTLV-2d based on the sequencing of the viral *env* gene and the 5′ long terminal repeat (LTR) region [5, 6, 15, 17, 28, 29].

HTLV-2 occurs with high prevalence among indigenous tribes of different linguistic families inhabiting the Brazilian Amazon, including in Kayapo villages, where subtype 2c was described for the first time [15, 17]. Later, its occurrence among the Kararaô, Gorotire, Tiriyo, Araral do laranjal and Zo’é indigenous populations was confirmed, and the same molecular subtype was found in urban populations of Brazil [6, 17, 30]. These results suggest that HTLV-2c is an endemic and autochthonous subtype in Brazil, particularly among indigenous populations of the Brazilian Amazon.

The geographical distribution of HTLV-2c in the Amazon region of Brazil ranges from the north (Tiriyo village) to the south (Kararaô and Gorotire villages), which suggests that its presence was derived during their settlement about 11,000 to 13,000 years ago during their migratory spread from the North to the South and the contact among different indigenous populations [6, 15]. In this context, the present study describes, for the first time, the occurrence of HTLV-2c and its dispersal in three new villages (Kateté, Djudjeko and Oodjã) of the Xikrin (Kayapo) ethnic group in the Brazilian Amazon region.

## Methods

### Study population and sample collection

**Fig. 1:**
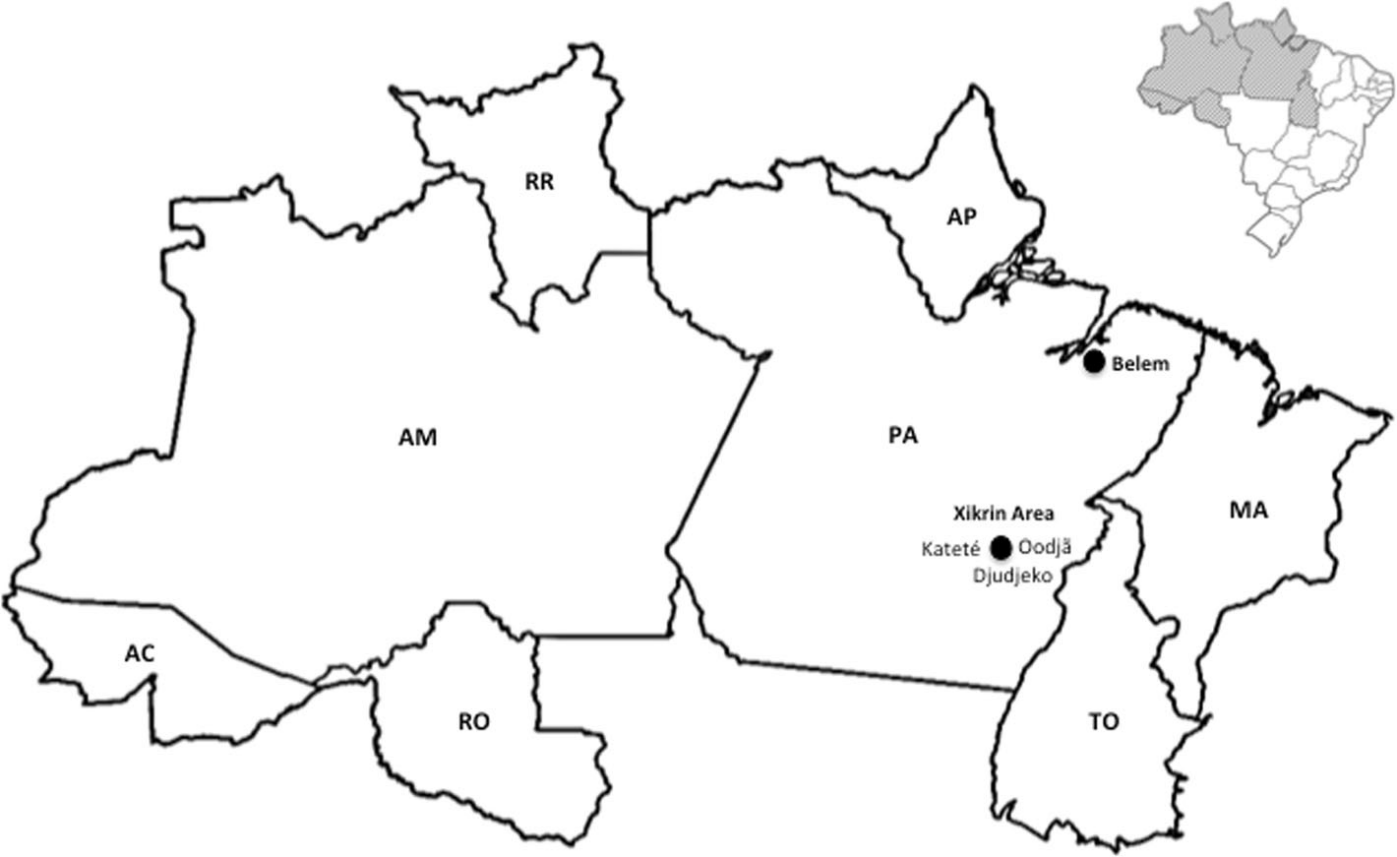
Map of the Brazilian Amazon region showing the geographic location of the Kateté, Djudjeko, Oodjã villages (Xikrin area) and Belem city, Capital of Para State (PA)

Blood samples (*n* = 263) were collected from individuals living in the Kateté (*n* = 121), Djujeko (*n* = 113) and Oodjã (*n* = 29) villages of the Xikrin tribe, Kayapo group, Jé linguistic family, who live in the Cateté Indigenous Territory, bordered by the Itacaiúnas and Cateté rivers, in the municipality of Parauapebas, state of Pará (Fig. 1). The study included individuals of both sexes aged 2–99 years old, from whom 10 mL of peripheral blood were collected in vacuum tubes containing ethylenediamine tetraacetic acid (EDTA) as anticoagulant for the separation of plasma and leukocytes. The samples were frozen at − 20 °C until subsequent analysis.

### Ethical considerations

The project was approved by the National Committee for Research Ethics (CONEP, process n° 961.451/2015). Following the CONEP’s recommendations, the project received, through formal written permission, the approval and consent of the communities through their leaders on behalf of the participants (adults and Children’s parents) and the National Indian Foundation (FUNAI), exempting individual consent in writing in accordance with national regulations.

### Serology

Plasma samples were tested for the presence of anti-HTLV-1/2 antibodies using an enzyme-linked immunosorbent assay (ELISA, Symbiosis Diagnostica LTDA, Leme, Brasil). The samples with positive or inconclusive results (presenting an optical density (OD) with values 20% below or above the cut-off value) were subjected to Real-Time PCR (Applied Biosystems, Foster City, CA, USA), for confirmation and differentiation between HTLV-1 and HTLV-2 infection.

### Real-time PCR (qPCR)

DNA from the seropositive and inconclusive samples was extracted according to the manufacturer’s protocol of the AxyPrep™*Blood Genomic DNA Miniprep* extraction kit (*Axygen Biosciences*, CA, USA) to amplify the *pol* and 5′ LTR regions. The qPCR reactions were prepared using the *TaqMan*® *Universal* master mix according to the following protocol: 10 μL of *MasterMix*, 4.0 μL of water, 1 μL of *Assay-by-Design (*primer and probe set) and 5 μL of DNA in a final volume of 20 μL. The following cycling protocol was used: one cycle of 50 °C for 2 min, 95 °C for 10 min and 40 cycles of 95 °C for 15 s and 60 °C for 1 min. Two target sequences, an endogenous control (human albumin gene) and the non-homologous regions of the *pol* gene (186 bp) of HTLV-1 and HTLV-2, were used. The primer sequences used were: 5′-gaacgctctaatggcattcttaaaaccc-3′(HTLV-1F), 5′-gtggttgattgattgtccatagggctat-3′ (HTLV-1R), 5′-caaccccaccagctacagg-3′ (HTLV-2F), 5′-gggcatgacaggttttgcaatatta-3′ (HTLV-2R), 5′-gctcaactccctattgctattcaca-3′ (Albumin F), 5′-gggcatgagaggttttgcaatatta-3′; and the probe sequences used were FAM-5′-acaaacccgacctaccc-3′-NFQ (HTLV-1), FAM-5′-tcgagagaaccaatggtataat-3′-NFB (HTLV-2) and FAM-5′-ttgtgggtgtaatcat-NFQ (Albumin).

### Nested PCR

Eight samples that were confirmed as positive for HTLV-2 were subjected to nested PCR reactions for amplification of the 5’LTR region for the later analysis of nucleotide sequencing and construction of phylogenetic trees. The 1st and 2nd round PCR reactions were run in a final volume of 50 μL containing 500 ng of extracted DNA, 10 μM of each dNTP, 20 pmol/μL of each primer, 50 μM MgCl2, 1 x buffer (50 mM KCl, 10 mM Tris-HCl pH 8.3) and 5 U of Taq DNA polymerase. In each amplification reaction, after initial denaturation at 94 °C for 5 min, 35 cycles were performed, with 40 s at 94 °C, followed by 30 s at 57 °C and 1 min at 72 °C. These 35 cycles were followed by a final extension for 10 min at 72 °C. The primers used were 5′-tcgcgatgacaatggcgactagcctc-3′ (F-IILTR) and 5′-gggggctttgggtattggagttggg-3′ (Long Gag) for the first round and 5′-gcctcccaagccagccac-3′ (Mo15) and 5′-gggaaagcccgtggatttgccccat-3′ (MSW-Gag) for the second round. The nested PCR products were visualized after electrophoresis (100 V/45 min) on 2% agarose gel in 1x TAE buffer (50x TAE stock solution - 1.6 M TrisBase, 0.8 M sodium acetate and 40 mM EDTA-Na2 in 1000 mL of deionized water) that contained 5 μL of ethidium bromide (10 mg/mL), using a transilluminator with an ultraviolet light source, and were subsequently purified using the manufacturer’s protocol of the QIAquick PCR Purification Kit (Qiagen, Inc., Valencia, CA, USA) for improving the nucleotide sequencing.

### Analysis of nucleotide sequences

The analyses of the degree of genetic divergence between the nucleotide sequences and the phylogenetic profile of the strains were performed using the MEGA-6 - Molecular Evolutionary Genetics Analysis 6.0 software [31].

### Phylogenetic analysis

The nucleotide sequences of the 5′ LTR region (642 nt) amplified in this study (DJU214 -MH194234, DJU218 - MH194235, OOD394 - MH194236, OOD396 - MH194237, KAT48 - MH194238, KAT147 - MH194239, KAT 116 - MH194240 and KAT46 - MH194241) were used to establish phylogenetic relationships with the HTLV-2 sequences that were previously described in the literature and are available in the GenBank. Sequence alignment was performed using the BioEdit software [32]. The degree of genetic diversity between the nucleotide sequences and the phylogenetic profile of the strains was analyzed using the MEGA-6 - Molecular Evolutionary Genetics Analysis 6.0 software [31]. The Neighbor-Joining method was used to construct the phylogenetic tree using the Kimura 2-parameters model. Statistical support of the phylogenetic tree was performed through bootstrap analysis, which generated 2000 replicates of the database.

## Results

### Serological findings

Antibodies to HTLV-1/2 were detected in 113 (43%) samples and 3 (1.1%) were undetermined (Table 1). The presence of HTLV-2 was confirmed by qPCR in 77 (29%) persons, with the following distribution: 46/121 (38%) from the Kateté village, 25/113 (22%) from the Djudjeko village and 6/29 (21%) from the Oodjã village. The indeterminate samples were negative on qPCR testing and no HTLV-1 infection was detected among samples tested.

**Table 1:** Prevalence of HTLV-2 infection in serum samples from three Xikrin villages (Kayapo) based on ELISA and qPCR testing

Communities	N	ELISA n (%)		qPCR n (%)
		Positive	Undetermined	Positive
Kateté	121	61 (50)	1 (0.8)	46 (38)
Djudjeko	113	45 (40)	2 (2)	25 (22)
Oodjã	29	7 (24)	0	6 (21)
Total	263	113 (43)	3 (1.1)	77 (29)

The prevalence rate according to sex was 38% in women and 18% in men (Table 2). This difference was observed in two communities. In the Djudjeko village, the values found were highly significant (*p* = 0.0030), corresponding to an infection prevalence of 32% in women and 8.5% in men. In the Kateté village, the percentage was 45% in women and 28% in men (*p* = 0.0609). In Oodjã, the prevalence rates (33 and 15%, respectively) were not statistically significant (*p* = 0.3391).

**Table 2:** Prevalence of HTLV-2 infection in the Xikrin villages (Kayapo), according to sex and age groups

Demographic characteristics	Kateté n (%)	Djudjeko n (%)	Oodjã n (%)
	Positive	Positive	Positive
Sex			
Female	32 (45)	21 (32)	3 (33)
Male	14 (28)	4 (8.5)	3 (15)
*p*	0.0609	0.0030	0.3391
Age group			
0–10	0	0	0
11–20	0	2 (10)	0
21–30	10 (26)	2 (8)	2 (25)
31–40	19 (54)	11 (32)	3 (43)
41–50	9 (47)	4 (22)	0
51–60	1 (20)	0	0
61–70	2 (33)	2 (50)	1 (50)
>70	5 (50)	4 (36)	0
*p*	0.0411	0.1786	0.3168
Total	46 (38)	25 (22)	6 (21)

A significant difference (*p* = 0.0005, Table 2) in HTLV-2 infection was observed when the cases were stratified by age. The prevalence was higher among individuals aged between 31 and 40 years (43%). No infection was observed in the 0- to 10-year-old age group. The difference according to age group was significant in the Kateté village (*p* = 0.0411).

A further association of infection with both age and sex was significant only in women (*p* = 0.0002, Table 3). The distribution of infection according to sex in each on age group showed significant differences between females and males in two groups aged 31–40 years (*p* = 0.017) and those over 70 years (*p* = 0.0019).

**Table 3:** Prevalence of HTLV-2 infection according to sex in each age group in Brazilian Amazon indigenous communities

Age group	Female		Male		*p*
	N	Positive (%)	N	Positive (%)	
0–10	2	0	1	0	1.000
11–20	20	2 (10)	13	0	0.507
21–30	36	7 (19)	36	7 (19)	1.000
31–40	48	26 (54)	28	7 (25)	0.017
41–50	21	8 (30)	18	5 (28)	0.734
51–60	3	1 (33)	4	0	0.428
61–70	6	4 (67)	6	1 (17)	0.242
> 70	10	8 (80)	11	1 (9.1)	0.002
*p*	0.0002		0.3385		

### Molecular characterization

Eight samples were subjected to amplification of a 788 bp segment of the 5′ LTR region for sequencing and subsequent phylogenetic analysis. The sequencing and editing of nucleotide bases generated a 642-bp fragment from the 5 ‘LTR region.

**Fig. 2:**
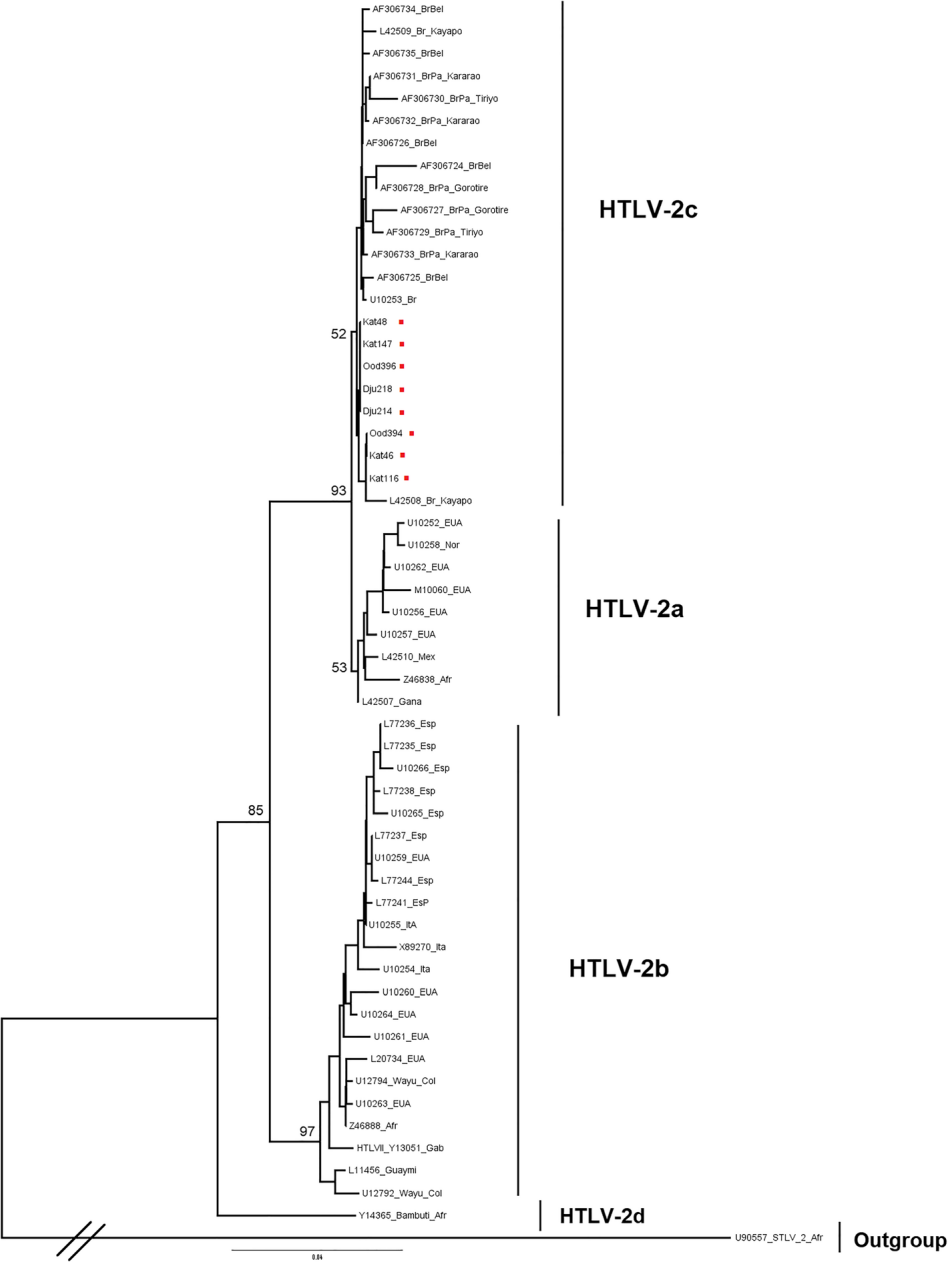
Rooted phylogenetic tree showing genetic relationships among the HTLV-2 strains of the samples identified in the present study (KAT46, KAT48, KAT116, KAT147, DJU214, DJU218, OOD394 and OOD396) and those available in the GenBank. STLV-2 strain (U90557-Afr) was used as outgroup. The tree was constructed using the Neighbor-Joining method after aligning the 642 nucleotides of the 5′ LTR region. The bootstrap test was applied using 2000 replicates

The sequences included samples from the three villages and were aligned and compared to each other, as well as with the prototype strains of subtypes 2a (HTLV-2^Mot.^) [33] and 2b (HTLV-2^NRA^) [34]. The samples from the present study presented 99.9% similarity to each other, and when compared to prototypes 2a and 2b, the identity between the sequences was 96,7% and 94,6%, respectively. The generated tree showed that the eight samples (KAT46, KAT48, KAT116, KAT147, DJU214, DJU218, OOD394 and OOD396) were clustered with the molecular subtype HTLV-2c as part of a monophyletic clade (Fig. 2).

## Discussion

In the present study the prevalence of 29% of HTLV-2 infection was found in three villages of the Xikrin tribe, Kayapo group: Kateté, Djujeko and Oodjã. The finding is consistent with previous results that demonstrated that the Kayapo group presents one of the highest endemic rates of HTLV-2 infection in the world [6, 11, 12, 14, 15].

Considering the results found for other indigenous peoples of Brazil, the prevalence rate found in the present study was higher. In the state of Paraná, in the Guarani group, the prevalence was previously reported as 5.8% [21]. More specifically, in the Brazilian Amazon region, lower rates were detected among the Kraho (12.2%), Mekranoiti (12.19%), Mundurukú (8.1%) and Macaw Laranjal Indians (11.4%) [11, 12, 15].

The prevalence found in the present study is similar to that observed in previous studies involving indigenous populations in the Americas. In Venezuela, the prevalence among the Guahibo [19] was estimated at 24.8%, and among the Seminole Indians in the United States, at 24% [24]. However, lower prevalence rates have been reported in the Yahgans in Chile (9.1%) [26], in the Wayuu in Colombia (4.8%) [35] and in the Wichi-Mataco in Argentina (above 3%) [25]. In two independent studies conducted in the Shipibo-Konibo group of the Peruvian Amazon [36], prevalence rates of 2.1 and 3.8% were found, but even lower prevalence rates were reported among the Nuu-Chah-Nulth of Canada (1.6%) and the Maya of Mexico (0.23%) [22, 27].

By contrast, the prevalence observed here was lower than those of several indigenous groups in the Americas. In Venezuela, the communities of Yaruro and Guahibo [20] had an estimated prevalence of 61%, and this rate is two times higher than that found in the present study. Higher prevalence rates were also found among the Chorote Indians in Argentina (35%), the Chulupi in Paraguay (34.0%) [18] and the Alcaf in Chile (34.8%) [26].

These differences in prevalence are expected and likely due to numerous factors, such as the: (i) differences in the methodologies adopted, which may have different sensitivities and specificities; (ii) differences in the sample sizes analyzed in each study; and (iii) differences in socio-cultural aspects that favor or limit the spread of the virus in the local population [6, 16]. Indigenous peoples, especially those in the Amazon, have undergone population fission and fusion events, which can contribute to increases or decreases in the incidence of the virus in a given community and often lead to the phenomenon known as the “founder effect”, where a small group of individuals (including virus carriers), due to internal conflicts over leadership, leave the tribe and start a new village. This situation, together with inbreeding, different women in the tribe breastfeeding children, polygamy and skin scarification rituals, may facilitate the interpersonal transmission of the virus and may consequently increase the prevalence of infection in the community [6]. These aspects may also explain the apparent increase in the prevalence of HTLV-2 infection in the Xikrin tribe when we compared our results with those of previous studies, which indicated a prevalence rate ranging from 13 to 15% [12, 13, 15].

It is generally agreed that HTLV infection is more easily transmitted from men to women, which would likely explain the higher prevalence of infection among women [18–20, 25, 37]. The first studies of HTLV-2 prevalence among the tribes of the Kayapo group also investigated the distribution of the infection according to gender [14, 15].

In the present study, there was a significant difference in infection prevalence between women and men, with the prevalence rate among women nearly twice the value in men. These results are in agreement with other findings in indigenous populations that found a predominance of infection among women. In the Peruvian Amazon, among the Shipibo-Konibo group, the reported seroprevalence was 3.1% in women and 2.5% in men [36]. In Venezuela, among the Yaruro and Guahibo, the prevalence of infection was 56% in men and 64% in women [20]. Among indigenous populations living in the Gran Chaco forest, the Chorote from Argentina and the Chulupi from Paraguay, there was a significant difference in infection between women (26.0%) and men (19.2%) [18]. Einsiedel et al. [38] reported, in central Australia, HTLV-1 infection associated with increasing age, male gender and sexual transmission infection in adults. The authors concluded that virus transmission from men to women was more efficient, which may also be suggested from the results of the present study.

However, the results also contrast with those of several studies conducted among indigenous groups in the Americas. Ishak et al. [15] did not observe differences in the prevalence rates between men (31.4%) and women (34.2%) when six tribes of the Kayapo indigenous groups were analyzed together. Similar results were observed in the Xikrin for women (11.4%) and men (11.7%) over the age of fourteen [14].

It is generally accepted that social and cultural characteristics are responsible to the differences of prevalence rates among native Indian communities, but these studies and the present results refer to three Kayapo Indians who share similar cultural behavior and common origin [14, 15]. For example, the hypothesized higher efficiency of male-to-female transmission commonly refers to the sexual route of transmission, and it is important to consider that this is not the only way by which the virus can be spread within a village.

Vertical transmission and breastfeeding are also common routes of spread among epidemiologically closed communities [6, 14, 15], which give support to the large dissemination of HTLV-2c among Kayapo children aged 0 to 9 years. It should be noted that in these villages, an infected lactating woman does not exclusively breastfeed her son or daughter, but she often feeds the offspring of other women from the tribe. In this sense, transmission via breastmilk could contribute to an equal distribution between the sexes. This was not described in the present study, which seems to suggest that a completely new behavior may have been introduced in the investigated communities. HTLV-2 is a persistent virus and the absence of infections among children 0 to 10 years (and in two groups up 20 years), may suggest that transmission of the virus is almost exclusively via sexual relations. The virus is apparently introduced in the community among young women who start to procreate in the second and third decades of life.

When the infection prevalence was analyzed by age group, a significant difference was observed between sexes. The highest prevalence was found in individuals between 31 and 40 years, and it was seven times higher than that observed in individuals aged 11 to 20 years and three times higher than that found in those aged 51–60 years. These findings are consistent with other studies conducted in other indigenous populations [15, 38]. Among the Toba, from Argentina, the prevalence found in individuals over forty years of age was 59%, which was four times higher than the infection rate in children (15.0%) younger than fifteen years [25]. In Amerindians living in the Gran Chaco forest in Argentina and Paraguay, the prevalence at 34 years was 26.3%, approximately twice as high as that found in children under the age of 13, whose prevalence rate was 14.2% [18]. In Venezuela, in the Guahibo group [19], there was an increase in infection prevalence with age, similar to that reported among the Kayapo [14, 15]. In the first study on the Kayapo, a prevalence of 60% was found in individuals over sixty years of age, approximately five times greater than that observed in children under fifteen years of age, which was estimated to be 12% [14]. In a second study among this group, conducted by Ishak et al. [15], the prevalence was 60% in individuals of both sexes older than 40 years and 21.6% in children under the age of nine. Thus, the results presented here, together with those of previous studies, reinforce the hypothesis that the sexual transmission of HTLV-2 is an important route of dissemination in the indigenous populations because its prevalence increases with age [14, 15, 18, 19].

The phylogenetic analysis is in agreement with previous studies that have demonstrated the endemic presence of HTLV-2, subtype c, in indigenous and urban populations of the Amazon [6, 15]. The samples formed a monophyletic clade with others that were previously identified as belonging to subtype 2c, thus confirming the isolated occurrence of this molecular subtype among the Kayapo.

A comparison of the nucleotide diversity of the 5’LTR region among the samples amplified in the present study identified a genetic similarity of 99.9% between them. When the analysis was performed considering the HTLV-2a Mot and HTLV-2b NRA prototype strains, the mean similarity was 96.7 and 94.6%, respectively. These results are similar to those observed among the Gorotire, Kararaô and Tiriyo indigenous communities and in the urban region of Belém [6]. Similarity of 99.4% was observed when comparing the nucleotide sequences of the LTR region between K96, isolated from the Kayapo Indians described as HTLV-2c and RP329 isolated from an inhabitant of the urban area in the state of São Paulo [37]. This subtype was also isolated in samples from HTLV-seropositive blood donors in the urban area of Porto Alegre, in the state of Rio Grande do Sul [30]. These results reveal the high degree of genetic similarity between the various HTLV-2c strains that have been isolated from Brazilian populations, which can be attributed to a unique, autochthonous origin and the miscegenation process that originated the neo-Brazilian population, as described recently by Vallinoto and Ishak [39].

## Conclusions

The results of the present study reveal the incidence of HTLV-2 in three new villages of the Xikrin tribe, thus confirming the high endemic nature of the infection in the Kayapo indigenous group of the Brazilian Amazon.

## Abbreviations

CONEP: National Committee for Ethics in Research
EDTA: Ethylenediamine tetraacetic acid
ELISA: Enzyme-linked immunosorbent assay
FUNAI: National Indian Foundation
HTLV-1: Human T-lymphotropic virus 1
HTLV-2: Human T-lymphotropic virus 2
LTR: Long terminal repeat
OD: Optical density
qPCR: Real-Time polymerase chain reaction
